# Embryonic diapause in humans: time to consider?

**DOI:** 10.1186/1477-7827-11-92

**Published:** 2013-09-17

**Authors:** Grazyna E Ptak, Jacek A Modlinski, Pasqualino Loi

**Affiliations:** 1 Department of Comparative Biomedical Sciences, University of Teramo, Piazza A. Moro, Teramo 64100, Italy; 2 Department of Experimental Embryology, Institute of Genetics and Animal Breeding of the Polish Academy of Sciences, Jastrzebiec, Poland

**Keywords:** Embryo, Delayed implantation, Embryonic diapause

## Abstract

**Background:**

When a competent blastocyst stage embryo finds itself in an unreceptive uterus, it delays development. In around one hundred species representing various orders, this delay is known to be reversible, but this phenomenon - termed embryonic diapause (ED) - is not considered a general characteristic of all mammals.

**Presentation of the hypothesis:**

Recently, however, we demonstrated that a non-diapausing species, the sheep, is capable of ED, suggesting the hypothesis that this is in fact an ancestral trait common to all mammals, including humans.

**Testing the hypothesis:**

In spite of the obvious difficulties in testing this idea, we propose a combination of indirect observations on human fertility patients, and direct study of the embryos of non-human primates.

**Implications of the hypothesis:**

Support for our hypothesis would require revision of obstetric interventions routinely performed when a human pregnancy extends beyond the due date.

## Background

Before implantation in the uterus, an embryo develops on its own. Once it reaches the blastocyst stage, however, its metabolism slows down naturally, and in the absence of appropriate stimuli from the uterus, it will not develop further [1]. The receptivity of the uterus may be blocked in response to unfavourable conditions for pregnancy, including metabolic, climatic or even psychosocial conditions [2-4]. In some mammalian orders, such as *Carnivora, Rodentia or Diprotodontia,* which often encounter harsh climate and face undernutrition or metabolic stress due to lactation, such situations occur quite frequently, and therefore the embryo present in the uterus enters diapause (Table 1). ED is a protective phenomenon. As such, it represents an important developmental advantage for species survival, and thus should be evolutionally maintained. Indeed, in other species (so called non-diapausing), an unreceptive uterus does not always result in the immediate failure of the blastocyst. In rodents [5], pigs [6], rabbits [7] and horses [8], for example, transferring the embryo into an unreceptive surrogate female can still result in a successful pregnancy. In spite of these observations, the phenomenon of diapause, in which an embryo stops development but remains viable for an extended period, was assumed to be rare in mammals. We recently demonstrated that diapause may be displayed by embryos of domestic sheep [9]. We were similarly successful in observing ED in rabbit and cow blastocysts following their transfer to unreceptive mouse uteri [Modlinski & Ptak, 2012, unpublished results]. Based on these observations, we hypothesize that ED is an ancient embryonic trait conserved across mammals, including humans. There are several lines of evidence indicating that this hypothesis needs to be taken seriously.

**Table 1:** Examples of embryonic diapause in placental mammals

***Taxonomy***		**Species**	**Gestation lenght**	**Embryonic diapause lenght**
*Order: Chiroptera*	Family: Pteropodidae	African fruit bat *(Eidolon helvum)*	7-8 months	3 -4 months
*Order: Carnivora*	Family: Mustelidae	Sable *(Martes zibellina*)	Up to 290 days	8 months
		Fisher (*Martes pennati*)	10-12	9-11 months
		North american river otter (*Lontra canadensis*)	10-13 months	8-11 months
		Short-tailed weasel (*Mustela erminea*)	9-10 months	8-9 months
		Long-tailed weasel (*Mustela frenata*)	8 months	7 months
		American mink (Neovison vison)	40-75 days	8-45 days
		American badger (*Taxidea taxus*)	8-9 months	6-7 months
		European badger (*Meles meles)*	4-12 months	2-10 months
	Family: Ursidae	Black bear (*Ursus americanus*)	7-8 months	5-6 months
		Brown bear (*Ursus arctos*)	8 months	6 months
		Giant panda (*Ailuropoda melanoleuca*)	100-150 days	50-100 days
	Superfamily: Pinnipedia	Northern fur seal (*Callorhinus ursinus*)	1 year	4 months
		Walrus (*Odobenus rosmarus*)	15-16 months	3-4 months
*Order: Cingulata*	Family: Dasypodidae	Nine-banded armadillo (*Dasypus novemcinctus*)	7-8 months	3-4 months
		Seven-banded armadillo (*Dasypus septemcinctus*)	6-8 months	2-4 months
*Order: Artiodactyla*	Family: Cervidae	Roe dear (*Capreolus capreolus*)	10 months	4-5 months

In animals, ED is usually the consequence of physiological stressors (photoperiod, lactation), while psychological stress is less likely to occur. In humans, conversely, the main cause of delayed implantation is related to psychological stress and smoking marijuana or nicotine [10-12]. All the aforementioned physiological and psychological stressors act through the hypothalamic-pituitary-gonadal (HPG) axis, leading to luteal dysfunction and, as a consequence, altering uterine receptivity, as well as through the hypothalamic-pituitary-adrenal (HPA) axis, directly blocking the receptivity of the uterus [4,10,12]. The mechanism of this block is similar in all species: high level of glucocorticoids is produced as a response to the stressor [13]. These hormones, in turn, mediate the down regulation of gonadotropins. Because gonadotropins are essential for the establishment of uterine receptivity, the implantation of the embryo is compromised whenever the maternal organism is under the action of the stressor [14-16].

In unreceptive human and mouse uteri, the concentration of anandamide (AEA) (an endogenous cannabinoid identified as a result of studies on the effects of Δ^9^-tetrahydrocannabinol, the active constituent of marijuana) increases sharply (reviewed in 10). As shown in a diapausing species, the mouse, as well as in a “non-diapausing” one, the sheep, AEA triggers reversible arrest of the embryo by binding to the cannabinoid receptors on the blastocyst [10,17]. AEA participates in the modulation of emotions and anxiety [18]. Therefore, augmented levels of cannabinoids in the human uterus, secreted in response to emotional stress (or due to smoking marijuana), may render implantation impossible. ED might be a natural consequence of such influences.

Delayed implantation was reported in humans long ago [19,20]. Wilcox *et al.* [1999] associated delayed implantation in humans with adverse pregnancy outcome, although the specific causes of pregnancy losses in their study were not evaluated [19]. Surprisingly and conversely to that human study, in animals delayed implantation appears to enhance the developmental potential of the embryo, as shown in rodents [5], pigs [6], rabbits [7] and sheep [21]. This increased developmental potential of mammalian embryos undergoing ED may be due to their cellular repair while being arrested (discussed in 9). Therefore, how can the increased number of miscarriages reported following delayed implantation in humans be explained [19]? We hypothesize that it is because those patients were also under stress *post* implantation. In such circumstances, the implanted conceptus, being unable to display ED any longer, is damaged by unfavourable uterine conditions, and the pregnancy will end in miscarriage. Such a situation is less likely to occur in animals, in which once lactation or a season unfavourable for pregnancy progression ends, the embryo develops without further disturbance. Notably, more recent human studies refuted the association of delayed implantation with pregnancy loss [22].

## Presentation and testing of the hypothesis

Although it has previously been suggested that ED might occur in humans [20,23], it has not been possible to test it experimentally, primarily because of ethical considerations. We propose two practical approaches that would allow this hypothesis to be explored further: indirectly, through an observational study on volunteer patients attempting to conceive (*Study 1*), and directly, using non-human primates (*Study 2*). A large-scale *Study 1* on human patients, conducted along the lines of earlier studies [19], would provide valuable data on the precise timing of implantation in relation to stress exposure. Patients would daily collect first urine specimens to identify the day of ovulation (estrogen and progesterone metabolites), and subsequently of implantation (hCG). Patients would also periodically provide blood samples to allow measurement of stress hormones. In addition, they would complete a detailed questionnaire in relation to stressors to which they are exposed, in the family or the work place. Stress exposure would be periodically evaluated until the third month of gestation. Pregnancy outcome will be analyzed at term, using routine neonatal tests (APGAR). Patients should be divided into 3 groups:

1) Delayed Implantation (≥11 days post ovulation), maternal stress confined to *pre* implantation, or no stress detected

2) Delayed Implantation (≥11 days post ovulation), maternal stress extended to *post* implantation

3) Implantation on time (days 8–10 post ovulation), no stress detected - control

The comparison of groups 1 and 3 will reveal if/how the delayed implantation influences the outcome of pregnancy. Furthermore, the comparison of groups 1 and 2 will allow the verification of if/how stress acting subsequently to delayed implantation may influence further pregnancy.

Direct confirmation of ED may be performed using non-human primates (*Study 2*), by studying the effects of a variety of experimentally induced stressors on implantation timing. In non-human primates (macaques or others) unreceptivity of the uterus should be induced during blastocyst development, expansion and attachment to the uterine wall (days 5–12 post coitus) by using endocannabinoids treatment (simulating marihuana smoking) or by inducing stress (for example social stress by overcrowding of animals). Then, similar to study 1 analysis of hormones level (in urine or in blood) should be performed in order to evaluate the day of implantation. A part of unimplanted diapausing blastocysts should be surgically flushed from the uterus and subjected to detailed analysis to improve our understanding of their molecular and functional characteristics. Other blastocysts should be allowed to implant and carried to term so as to evaluate the consequences of ED on foetal development.

## Implications of the hypothesis

If based on a reasonably high number of pregnancies, both studies (*1* and *2*) will address not only whether ED is a feature of human pregnancy, but also whether it is beneficial or harmful for pregnancy progression. If human embryo is able to enter diapause, this has huge implications for human pregnancy. If proven, the existence of human embryo diapause could question the adequacy of common gynecological practices, such as the estimation of the gestational age based on the last menstrual cycle or the stimulation of parturition. This hypothesis, if confirmed, would have a great impact on human reproductive medicine, opening up a fertile new investigation into the benefits or otherwise of ED in humans. Accordingly, common obstetric definitions, such as “foetus small for gestational age” or “prolonged pregnancy” may need to be revised. If ED occurs in humans, the current arbitrary use of day of last menstrual period to estimate ovulation and pregnancy duration will inevitably be misleading. The measure of effective date of implantation through a simple pharmaceutical device similar to those that currently measure hCG concentration would be of immense value, helping to reduce the uncertainty of overdue dates and to prevent unnecessary surgical interventions. Such tests should be done at least in those pregnancies in which the risk of complication or of reduced foetal growth is high, such as those obtained by assisted reproduction. Furthermore, if the developmental potential of the embryo may be enhanced by ED, undergoing diapause by embryos obtained by assisted reproduction (for example by their asynchronous transfer) may be suggested as a therapeutic approach. Furthermore, from the socio-economic point, the knowledge about this phenomenon may change cultural life-styles of the couples particularly around the time of embryo conception and implantation. Indeed the importance of the maternal contribution to the first phase of pregnancy (i.e. to periimplantation period) are often underestimated.

## Competing interests

The authors declare that they have no competing interests.

## Authors’ contributions

GEP concived the study and wrote the manuscript. JAM and PL participated in its design and helped to draft the manuscript. All authors read and approved the final manuscript.
